# Molecular Modeling of Subtype-Specific Tat Protein Signatures to Predict Tat-TAR Interactions That May Be Involved in HIV-Associated Neurocognitive Disorders

**DOI:** 10.3389/fmicb.2022.866611

**Published:** 2022-04-07

**Authors:** Monray E. Williams, Ruben Cloete

**Affiliations:** ^1^Human Metabolomics, North-West University, Potchefstroom, South Africa; ^2^South African Medical Research Council Bioinformatics Unit, South African National Bioinformatics Institute, University of the Western Cape, Bellville, South Africa

**Keywords:** HIV-associated neurocognitive disorders, Tat polymorphisms, molecular modeling, molecular docking, molecular dynamic simulation

## Abstract

HIV-1 is responsible for a spectrum of neurocognitive deficits defined as HIV-associated neurocognitive disorders (HAND). The HIV transactivator of transcription (Tat) protein plays a key role in the neuropathophysiology of HAND. The Tat protein functions by transactivation of viral genes through its interaction with the transactivation response (TAR) RNA element. Subtype-specific Tat protein signatures including C31S, R57S and Q63E present in Tat subtype C has previously been linked to a lowered neuropathophysiology compared to Tat subtype B. In this study, we attempted to understand the molecular mechanism by which Tat subtype-specific variation, particularly, C31S, R57S, and Q63E influence the Tat-TAR interaction. We performed molecular modeling to generate accurate three-dimensional protein structures of the HIV-1 Tat subtypes C and B using the Swiss model webserver. Thereafter, we performed a molecular docking of the TAR RNA element to each of the Tat subtypes B and C protein structures using the HDOCK webserver. Our findings indicate that Tat subtype B had a higher affinity for the TAR RNA element compared to Tat subtype C based on a higher docking score of −187.37, a higher binding free energy value of −9834.63 ± 216.17 kJ/mol, and a higher number of protein–nucleotide interactions of 26. Furthermore, Tat subtype B displayed more flexible regions when bound to the TAR element and this flexibility could account for the stronger affinity of Tat subtype B to TAR. From the Tat signatures linked to neuropathogenesis, only R57/R57S are involved in Tat-TAR interaction. Due to the lack of electrostatic interactions observed between Tat subtype C and TAR, weaker affinity is observed, and this may contribute to a lower level of neuropathophysiology observed in subtype C infection.

## Introduction

The effects of HIV-1 within the immune system have been well established, however, HIV-1 is also responsible for causing dysfunction of the central nervous system (CNS; González-Scarano and Martín-García, 2005). Regardless of viral suppression, approximately 50% of the HIV-1 population may continue to present with a spectrum of neurocognitive impairments defined as HIV-associated neurocognitive disorders (HAND; Antinori et al., 2007; Heaton et al., 2011).

The prevalence of HAND and clinical severity have been linked to HIV-1 subtype variation (Rao et al., 2008, 2013). HIV-1 is divided into four groups including M, N, O, and P. Group M is considered the “major” group responsible for the global human HIV epidemic and this group is subdivided into nine subtypes (A, B, C, D, F, G, H, J, and K) and at least 51 circulating recombinant forms (CRFs; de Arellano et al., 2010; Hemelaar, 2012). The majority of the understanding of the neuropathogenesis of HIV-1 is derived from studies of HIV-1 subtype B (HIV-1B) which is present in America, Western Europe, and Australia and represents about 12% of all HIV infections (Geretti, 2006; Taylor et al., 2008; Tyor et al., 2013). In contrast, the dominant HIV-1 subtype C (HIV-1C) is responsible for the highest HIV-1 prevalence (>50% of cases) and is present in countries of Southern Africa and India (Geretti, 2006; Shen et al., 2011). The neuropathophysiology related to the onset of HAND are different between HIV-1B and HIV-1C (Tyor et al., 2013; Santerre et al., 2019). In particular, the subtype-specific differences can be linked to sequence variation within key viral proteins including glycoprotein 120 (Colon and Vazquez-Santiago, 2015), Viral protein R (Dampier et al., 2017), and transactivator of transcription (Tat; Rao et al., 2008).

The Tat protein is of particular interest due to its multifunctional activity within the CNS as well as its persistent expression despite the use of ART (Mediouni et al., 2012). Tat functions in HIV viral transcription from a long terminal repeat (LTR) promoter *via* interaction with the transactivation response (TAR) element sequence at the 5’ end of the LTR (+1 to 59+; Dingwall et al., 1989). TAR forms a stable stem-loop structure in which a key element is a 3-nucleotide bulge (UCU; position 23–25; Roy et al., 1990; Davidson et al., 2009). Tat binds directly to this bulged region (Berkhout et al., 1989; Selby et al., 1989) for the transactivation of viral genes. The loop region in TAR (position 30–35) is also required for transactivation (Aboul-ela et al., 1996; Wemmer, 1996). With regards to Tat, the N-terminal region is not directly involved in TAR interaction, however, it is required for viral transactivation (Demarchi et al., 1999). The key Tat residues which are largely responsible for TAR interaction include the basic region of nine residues, in which arginine residues mediates the specific recognition of TAR (Demarchi et al., 1999). Tat is known to interact with multiple host factors that ensure the binding affinity of Tat to TAR, however, we focused particularly on the major interacting partner which is TAR to understand how Tat variants result in varying levels of binding.

When comparing Tat subtype B and subtype C in its ability to bind TAR and transactivate, the findings are mixed, with studies suggesting Tat subtype C to have a more flexible structure and this allows for effective binding to partners resulting in increased transactivation (Johri et al., 2015). The inverse has argued that Tat subtype B has greater flexibility with greater binding and transactivation capacity (Siddappa et al., 2006; Ruiz et al., 2019). Further, Tat-specific signatures are related to differential Tat-TAR interaction and/or transactivation. The Arginine’s 52 and 56 were key for rigid TAR-Tat complex formation, while the other C-terminal Arginine’s R53, R55, and R57 contribute to specific binding to a lesser extent (Edwards et al., 2005). Tat peptides with an arginine residue at position 56 appear to consistently perform 2- to 3-fold better in transcription activation studies than peptides with lysine or another amino acid at this position (Xie et al., 2003, 2004). Further, a Q63E mutation present in Tat subtype C was shown to contribute to higher transcriptional activation in human CD4 T cells (Kurosu et al., 2002).

It has not been established whether Tat protein signatures related to the neuropathophysiology of HIV-1 may affect Tat-TAR interactions. In a previous review done by our group, Tat protein signatures C31S, R57S, and Q63E present in Tat subtype C were reported to differentially affect mechanisms related to the development of HAND (Williams et al., 2020). In addition to the neuropathogenic effects of these Tat signatures, here we investigated whether these signatures influenced Tat-TAR interaction between subtype B and C. Therefore, this study aimed to determine (1) which Tat subtype had the highest binding affinity for TAR, (2) which subtype-specific Tat residues were crucial for TAR interaction, and (3) which residues from the neuropathogenic subtype-specific Tat protein signatures (at position 31, 57, and 63) may be important in Tat-TAR interaction.

## Materials and Methods

### Retrieval of HIV-1 Subtype B and Subtype C Tat Sequences and the TAR RNA Structure

The HIV-1 subtype B and subtype C Tat protein sequences were retrieved from the Universal Protein Databases (UniProt). We used the HIV Tat subtype B (Isolate MN)^1^ and HIV Tat subtype C (Isolate 92BR025)^2^ as these contained the sequence variations related to the differential HIV-1 neuropathogenesis (Williams et al., 2020). The experimentally solved 3D structure for the TAR RNA was downloaded from the protein data bank (PDB ID: 1ANR). The region of TAR (from 17 to 45 nucleotides) which encompasses the bulge (+23 to +25) was used in this study as it contains the known interacting nucleotides that bind to HIV-1 Tat protein (Dingwall et al., 1989).

### Sequence Alignment

We conducted a pairwise sequence alignment between HIV Tat subtype B and HIV Tat subtype C using MAFFT version 7 which does both pairwise and multiple alignment of amino acid or nucleotide sequences. The result of the pairwise sequence alignment was visualized using Jalview to identify all possible sequence variations between Tat subtype B and Tat subtype C.

### Secondary Structure Prediction

Secondary structure elements consisting of alpha-helices, beta-sheets, and random coils were predicted for HIV Tat subtype B and HIV Tat subtype C using the PSIPRED secondary structure prediction server^3^ (McGuffin et al., 2000). Secondary structure prediction is an important first step toward tertiary structure prediction, as well as providing information about protein activity, relationships, and functions (Ma et al., 2018).

### Prediction of Disordered State of Tat Variants

The DISOPRED3 program was used for protein disorder prediction and for protein-binding site annotation within disordered regions available at http://bioinf.cs.ucl.ac.uk/disopred (Jones and Cozzetto, 2015). The server allows users to submit a protein sequence and returns a probability estimate of each residue in the sequence being disordered. Briefly, Tat protein sequences for each variant was uploaded to the database for residue disorder prediction.

### 3D Structure Prediction Using Swiss Model

Swiss model^4^ is a fully automated protein structure homology-modeling server (Schwede et al., 2003; Waterhouse et al., 2018) which was used to model Tat subtype B and Tat subtype C. The respective Tat protein sequences were used as input to the Swiss model web interface. From the input sequence, Swiss model does (1) template search, (2) template selection and alignment, (3) model building, and (4) model quality assessment (Schwede et al., 2003; Waterhouse et al., 2018). For the prediction of Tat subtype B, the template ID: 1JFW.1.A (sequence identity: 90.7% and coverage: 0.85) and for Tat subtype C, the template ID: 1TBC.1.A (sequence identity 72.09 and coverage: 0.85) were selected. The templates above had the highest sequence identity and coverage to the target sequences and were selected for model building. The Swiss model webserver reports in built quality assessment scores for protein models predicted using the webserver, such as the Global Model Quality Estimate (GMQE) score (Biasini et al., 2014). The GMQE score gives an overall model quality measurement between 0 and 1, with higher numbers indicating higher accuracy of the model built with that specific alignment and template (Biasini et al., 2014).

### 3D Structure Quality Assessment

To assess the quality of the predicted 3D structures, a variety of structural parameters were tested within each model. Procheck from the Structural Analysis and Verification Server (SAVES)^5^ was used to determine if the predicted residues were within the allowable region of the Ramachandran plot (Laskowski et al., 1993). Structures were considered reliable if the majority (>80%) of residues had favorable phi and psi dihedral angle distributions. ProSA-web-Protein Structure Analysis^6^ was used for the recognition of errors in three-dimensional structures of proteins and to measure total energy deviation within the protein structure (Wiederstein and Sippl, 2007). This was used to determine whether the *z*-score of the input structure is within the range of scores typically found for native proteins of similar size. Lastly, the root mean square deviation (RMSD) values were calculated between the predicted structure and the homologous template structure using PYMOL/Maestro molecular visualizing software to compare backbone structural similarity to the experimentally solved template structure. Highly similar structures are considered when the RMSD is below 2 Å suggesting homology (Chothia and Lesk, 1986) whereas higher RMSD indicates that predicted structures and templates are not structurally similar. Protein structures that satisfy most or all of the quality parameter tests are considered reliable for subsequent docking studies.

### Refinement and Energy Minimization

The predicted 3D structures were subsequently energy-minimized with 3Drefine^7^ which refines the structure by minimizing atomic-level energy and optimizing hydrogen bonding network and reduces steric clashes between atoms (Bhattacharya et al., 2016). The force field consists of a combination of physics-based and knowledge-based terms which involves the optimization of hydrogen bonding networks combined with atomic-level energy minimization on the optimized model using a composite physics and knowledge-based force field. The physics-based terms include the energetic contributions of the bonded interactions described in the Energy Calculation and Dynamics potential (Levitt et al., 1995; bond length, bond angle, and torsion angle) along with a tethering term of the Cα and Cβ atoms (Bhattacharya and Cheng, 2013). The knowledge-based terms include the atomic pairwise potential of mean force (Summa and Levitt, 2007) and explicit hydrogen bonding potential. The final energy-minimized model is the lowest energy minima conformation of the protein structure.

### Molecular Docking

The docking of the TAR element to the Tat proteins were carried out using HDOCK server, a free online web server that enables the docking of the protein–RNA molecules based on a hybrid algorithm of template-based modeling and *ab initio* free docking, available at http://hdock.phys.hust.edu.cn/ (Yan et al., 2020). Moreover, HDOCK also supports protein–RNA/DNA docking with an intrinsic scoring function. In brief, the 3D minimized structures of the respective Tat proteins and the TAR structure retrieved from PDB were uploaded to the HDOCK server. The basic region of the Tat protein (residues 48–58; Calnan et al., 1991; Weeks and Crothers, 1991; Ronsard et al., 2017) and the bulge region of the TAR (+23 to +25; Dingwall et al., 1989) is the known Tat-TAR binding site and therefore these were given as input active site residues to specify the search space for the docking simulation. As a validation step, blind docking was performed to determine if the correct binding site was specified.

### Protein–RNA Interaction Analysis

Protein–RNA interaction analysis was done using the protein–ligand interaction profiler (PLIP; Adasme et al., 2021).^8^ Briefly, the docked complexes of Tat subtype B/subtype C and TAR were uploaded to the PLIP webserver. PLIP detects hydrogen bonds, hydrophobic contacts, π-stacking, π-cation interactions, salt bridges, water bridges, metal complexes, and halogen bonds between ligands and targets. Cut off for interactions formed were 4.1 Å for hydrogen bonds, 4.0 Å for hydrophobic contacts, 5.5 Å for π-stacking, 6.0 Å for π-cation interactions, 5.5 Å for salt bridges, 4.1 Å for water bridges, 3.0 Å for metal complexes, and 4.0 Å for halogen bonds.

### Molecular Dynamic Simulations

Two simulation systems consisting of Tat subtype B-TAR and Tat subtype C-TAR complexes were prepared using the CHARMM-GUI webserver (Jo et al., 2008; Lee et al., 2016). Both systems were solvated with TIP3 water molecules in a cubic box of at least 10 Å of water between the protein and edges of the box at a concentration of 0.15 M. To neutralize the positive and negative charges of the systems for Tat subtype B-TAR, 43 potassium (K) ions and 26 chloride (Cl) ions were added to neutralize the charge of the system while the Tat subtype C-TAR system had 53 K ions and 30 Cl ions, respectively.

Each system underwent 50,000 steps of steepest descents energy minimization to remove steric overlap. Subsequently, both systems were subjected to a two-step equilibration phase, namely, NVT (constant number of particles, Volume and Temperature) for 100 ps to stabilize the temperature of the system and a short position restraint NPT (constant number of particles, pressure, and temperature) for 500 ps to stabilize the pressure of the system by relaxing the system and keeping the protein restrained. For the NVT simulation, the system was gradually heated by switching on the water bath and the V-rescale temperature-coupling method was used, with constant coupling of 0.1 ps at 300 K under a random sampling seed. While for NPT the Parrinello–Rahman pressure coupling (Parrinello and Rahman, 1981) was turned on with constant coupling of 0.1 ps at 300 K under conditions of position restraints (all-bonds). For both NVT and NPT, electrostatic forces were calculated using the Particle Mesh Ewald method (Essmann et al., 1995). Both systems were subjected to a full 50 ns simulation using the GROMACS-2019 package (Abraham et al., 2015) along with the CHARMM36M all-atom force field (Huang et al., 2017). The analysis of the trajectory files was done using GROMACS utilities. The root mean square deviation (RMSD) was calculated using gmx rmsd for the protein back bone atoms and the TAR heavy chain atoms, while the root mean square fluctuation (RMSF) for the protein residues were calculated using gmx rms. The average number of hydrogen bonds formed between the Tat subtype C protein and the TAR element was calculated using the gmx hbond tool. The free energy of binding was calculated using the Molecular Mechanics Poisson–Boltzmann Surface Area (MMPBSA) protocol implemented in g_mmpbsa package over the last 500 frames of the simulation trajectory (Kumari et al., 2014).

### Protein–RNA Hotspot Residues

Prediction of protein–RNA binding hot spots (PrabHot) webserver^9^ is an online tool that is used for the prediction of residue hotspots in protein–RNA interfaces using an ensemble approach (Pan et al., 2018). Residues or cluster of residues that make a major contribution to the binding free energy within an interaction is considered hotspot residues (Zerbe et al., 2012). Briefly, the Tat subtype B-TAR and Tat subtype C-TAR docking files were uploaded to the PrabHot webserver. First, hotspot residues were determined for the interaction between Tat subtype B and TAR (as the known positive control). This was done to determine if certain Tat subtype B residues were crucial for the interaction with TAR and to determine if these predicted hotspot residues were mutated and present in Tat subtype C. Thereafter, hotspot residues were determined for the interaction between Tat subtype C and TAR.

## Results

### Sequence Alignment

**Figure 1:**
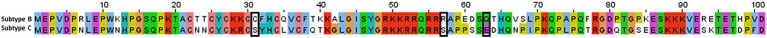
Pairwise sequence alignment between Tat Subtype B (top) and Tat Subtype C (bottom). Key sequence variants, C31S, R57S, and Q63E are shown within boxes.

Percentage sequence identity between Tat Subtype B and subtype C was = 72.28% and the pairwise alignment score between Tat Subtype B and subtype C was 4160.0 (Figure 1). Several sequence variations exist between Tat Subtype B and subtype C, however key sequence variants with a reported effect on neurocognitive outcomes include C31S, R57S, Q63E.

### Secondary and Tertiary Structure Prediction and Quality Assessments

**Figure 2:**
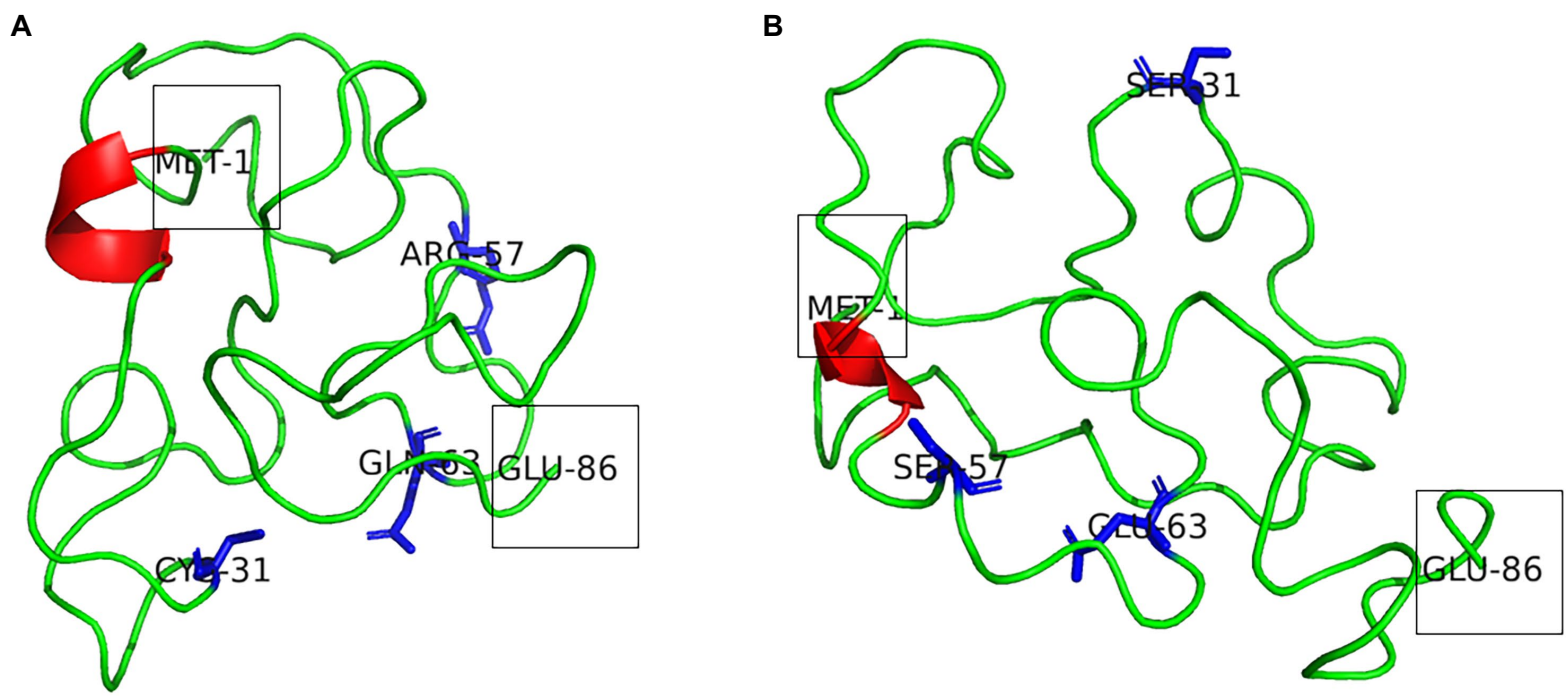
**(A)** Model of Tat subtype B and **(B)** Tat subtype C. The alpha-helical structure is indicated in red. The N-terminal Met1 and C-terminal Glu86 are shown within boxes. The Tat variants at positions 31 (Cys31/Ser31), 57 (R57/S57), and 63 (Q63/E63) are shown as blue sticks.

Secondary structure prediction of Tat subtype B and Tat subtype C from PSIPRED indicate that both proteins adopted a three alpha-helical structure (Supplementary Figure 1). However, findings from the 3D structures indicate that both Tat subtype B and subtype C had one alpha-helical structure within the core and basic domain, respectively (Figures 2A,B). Both Tat variants had disordered states between amino acids 51–73 and 92–95 (Supplementary Figure 2A; Figure 2B). Tat subtype C (26) had a higher number of disordered residues compared to Tat subtype B (25). Furthermore, the 3D predicted Tat subtype B and C protein structures had high GMQE indicating that the models were of reliable quality and accuracy (Table 1). Both Tat models successfully passed Procheck assessment as >80% of the residues were within the allowable regions of the Ramachandran plot (Table 1). ProSA analysis indicated that both structures *z*-scores were within the range of scores typically found for native proteins of a similar size (Table 1). Furthermore, the RMSD scores for the Tat proteins were less than 2 Å when compared to the homologous templates suggesting high structural similarity (Table 1; Supplementary Figures 3A,B). The predicted 3D structures of Tat subtype B and C satisfied all the quality parameter tests and were considered for subsequent docking studies.

**Table 1:** Summary of the quality assessment scores for the 3D predicted structures of Tat subtype B and C.

Tat subtype	Template	GMQE	Procheck (percentage in allowed region)	ProSA (*z*-score)	RMSD (Å)
B	1jfw.1.A	0.50	Pass (88.3%)	Pass (−3.05)	0.169
C	1tbc.1.A	0.45	Pass (98.5%)	Pass (−1.36)	0.515

GMQE, Global Model Quality Estimate; ProSA, protein structure analysis; and RMSD, root mean square deviation.

### Molecular Docking: HDOCK

Most residues within the basic region (residues 48–58) of Tat were found to interact with the TAR element for both Tat subtypes (Table 2). Several types of interactions were identified between Tat and the TAR element which included hydrogen bonds (h-bonds), salt bridges, hydrophobic and π-Cation interactions. Tat subtype B reported a docking score of −187.37 with a total number of 26 interactions with TAR, which consisted of 21 Hydrogen bonds (H-bonds) and five salt bridge interactions (Table 2). On the other hand, Tat subtype C reported a docking score of −174.45 with a fewer total number of interactions of 13 which included 10 H-bonds and one salt bridge, one pi-stacking, and one hydrophobic interaction (Table 3). Tat Subtype B had a greater number of interacting residues of 16 with TAR compared to Tat subtype C having nine. From the key protein signatures related to neurocognitive outcomes (Williams et al., 2020), only Arg57 in Tat subtype B and Ser57 in Tat subtype C were interacting with TAR.

**Table 2:** The number and type of interactions for both Tat subtype B and C bound to TAR.

Protein	H-Bonds (nucleotides)	Salt bridge (nucleotides)	π-Cation (nucleotides)	Hydrophobic (nucleotides)
Tat subtype B	Met1 (U38), Glu2 (A22, U23), Asp5 (C19), Ser16 (G36, G36), Cys22 (A35), Ser46 (C37, C37), Tyr47 (G46), Lys 50 (U25, A27, G26), Lys51 (A22), Arg53 (U23, A22, U42), Gln54 (A20), Arg55 (A20), Arg56 (C19), and Arg57 (C19)	Lys19 (G36), Lys40 (G34, G35), and Arg55 (C19, G18)	–	–
Tat subtype C	Gln39 (C24), Ser46 (G33, G33), Gly48 (G34, U25), Lys50 (U25), Arg52 (C24, G21), Arg55 (C24), and Ser57 (A20)	HIS33 (G36)	Lys50 (U25)	TYR47 (U25)

**Table 3:** Molecular Mechanics Poisson–Boltzmann Surface Area (MMPBSA) energy parameter contributions to the total binding free energy.

Protein	Van der Waals energy (kJ/mol)	Electrostatic energy (kJ/mol)	Polar solvation energy (kJ/mol)	Solvent accessible surface area (SASA) energy (kJ/mol)	Total ΔG bind protein-5RE (kJ/mol)
Tat subtype B-TAR	−286.58 ± 40.47	−11475.25 ± 291.47	1970.38 ± 197.41	−43.19 ± 3.74	−9834.63 ± 216.17
Tat subtype C-TAR	−267.06 ± 29.07	−8735.39 ± 211.95	2078.52 ± 183.37	−41.35 ± 3.40	−6965.28 ± 229.24

**Figure 3:**
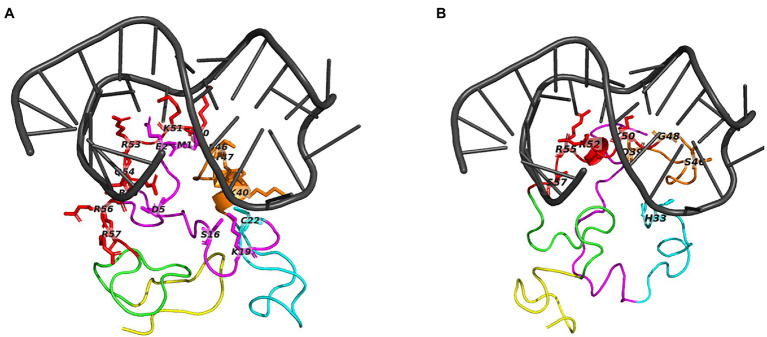
The top predicted binding pose for TAR docked to Tat subtype B and C. Panel **(A)** shows TAR bound to Tat subtype B and **(B)** TAR bound to Tat Subtype C. Molecular docking was carried out with the basic region of Tat (cartoon structure) with the bulge region of TAR (grey). Tat is presented as a coiled cartoon structure, with domains represented including the proline-rich region (magenta), cysteine-rich (cyan), core (orange), arginine-rich (red), glutamine-rich (green), and the RGD domain (yellow). Interacting residues are presented as the single letter code. The interacting nucleotides are presented in Table 2.

Both Tat subtypes are bound to the same binding pocket respectively, regardless of selecting active site residues or performing a blind docking (Supplementary Figure 4). The blind docking of TAR to Tat subtype C had a slight change in TAR structure (Supplementary Figure 4). Tat subtype B and subtype C interacted with TAR at similar binding sites (Figures 3A,B); however, Tat subtype B had a great number of interactions (Figure 3A) when compared to Tat subtype C (Figure 3B). The majority of the interacting residues were located in the Arginine-rich domain (Figure 3-red) for both Tat subtype B and C.

### MD Simulations and MMPBSA Analysis

**Figure 4:**
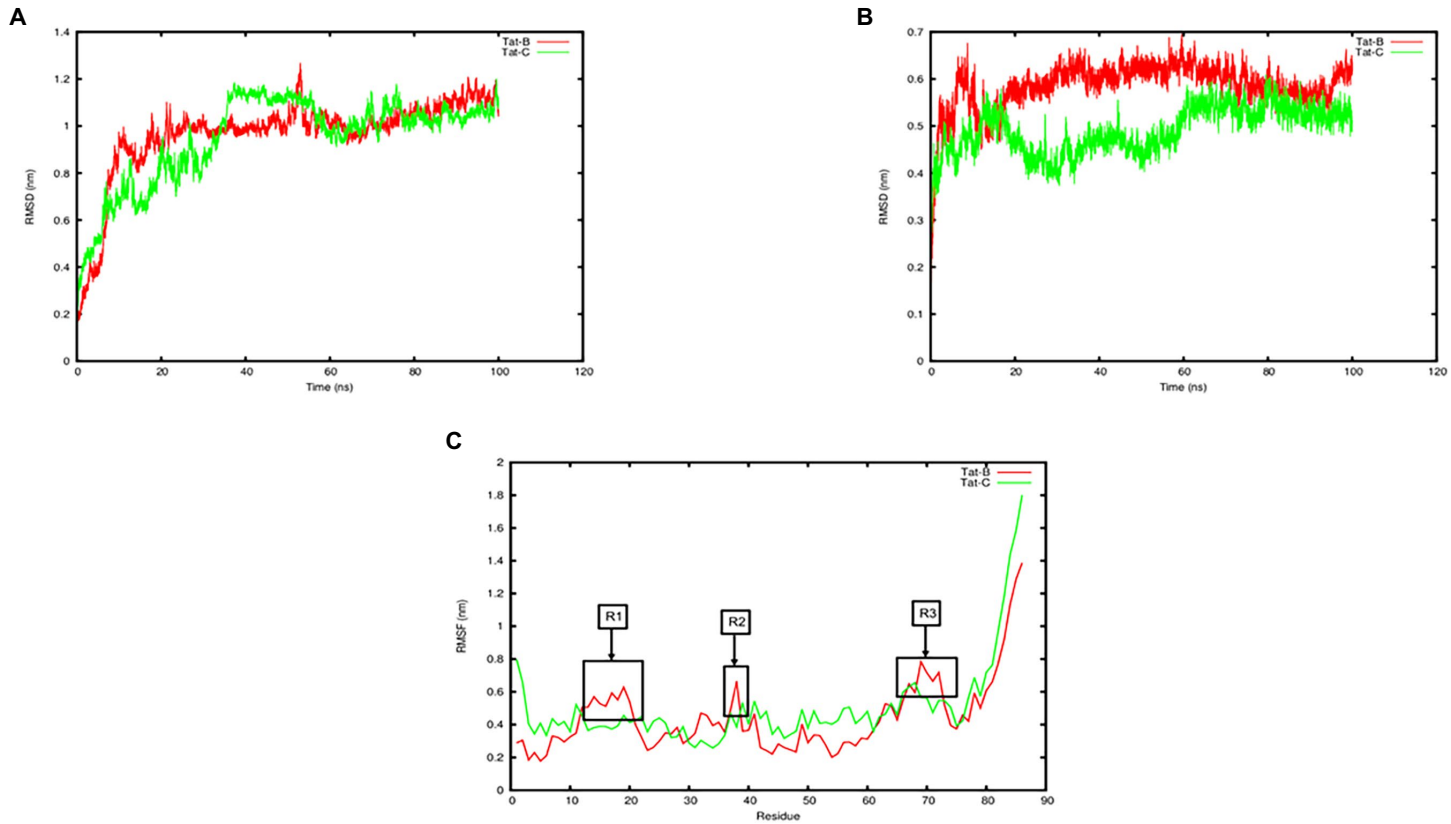
Plot of molecular dynamic simulation trajectories of Tat subtype B and Tat subtype C interaction with TAR. **(A)** Tat subtype B-TAR and Tat subtype C-TAR systems on the backbone RMSD values over 100ns. **(B)** TAR element subtype B and TAR element subtype C systems heavy chain atoms RMSD values over 100ns. **(C)** Root mean square fluctuation (RMSF) of the protein residues between Tat subtype B-TAR and Tat subtype C-TAR. Highly flexible regions R1–R3 are labelled and shown in boxes.

Tat subtype B-TAR and Tat subtype C-TAR systems both reached equilibrium after 60 ns based on the backbone RMSD values (Figure 4A). The mean and SD values for the change in protein RMSD backbone atoms for Tat subtype B-TAR and Tat subtype C-TAR were 0.96 ± 0.18 nm and 0.94 ± 0.19 nm, respectively (Figure 4A). Furthermore, the RMSD values for the heavy chain atoms of the TAR element of Tat subtype C were lower at 0.49 ± 0.05 nm compared to the TAR element of Tat subtype B reaching 0.58 ± 0.05 nm (Figure 4B). The RMSF fluctuation values for the protein residues were the lowest for Tat subtype B-TAR, having 0.44 ± 0.22 nm compared to Tat subtype C-TAR with the largest RMSF value of 0.50 ± 0.26 nm (Figure 4C). The protein residues of Tat subtype B showed three regions (residues R1: 13–20, R2: 37–38, and R3: 68–72), of high flexibility, compared to Tat subtype C with no regions of high flexibility (Figure 4B). None of the active site residues (residues 48–58) showed high flexibility values, suggesting the active site is stable and not undergoing large conformational changes (Figure 4B). However, two contact residues S16 and K19 from Tat subtype B in Region 1 showed higher flexibility values compared to Tat subtype C (Figure 4C). Interestingly, the average number of hydrogen bonds formed between Tat subtype C and TAR was the highest with 14.02 contacts being formed compared to Tat subtype B and TAR having 10.47 bonds, respectively. However, the total binding free energy calculated for Tat subtype B-TAR was the highest of −9834.63 ± 216.17 kJ/Mol in comparison to Tat subtype C-TAR having a total binding free energy score of −6965.28 ± 229.24 kJ/Mol (Table 4).

**Table 4:** Prediction of Protein–RNA binding energy hot spots between Tat subtype B and C and TAR.

Protein	Hotspot residues (score)
Tat subtype B	Met1 (0.84), Glu2 (0.58), Val4 (0.84), Asp5 (0.82), Lys19 (0.81), Thr20 (0.51), Ala21 (0.84), Cys22 (083), Gln35 (0.54), Lys40 (0.54), Ile45 (0.83), Ser46 (0.82), Tyr47 (0.83), Gly48 (0.77), Arg49 (0.79), Lys50 (0.83), Lys51 (0.83), Arg52 (0.81), Arg53 (0.82), Gln54 (0.82), Arg55 (0.82), Arg56 (0.81), and Arg57 (0.79)
Tat subtype C	Glu2 (0.82), His33 (0.81), Thr40 (0.84), Gly44 (0.82), Ser46 (0.82), Tyr47 (0.84), Gly48 (0.79), Arg49 (0.82), Lys50 (0.79), Lys51 (0.81), Arg52 (0.81), Arg55 (0.79). Arg56 (0.79), Ser57 (0.79), Ala58 (0.81), and Pro59 (082)

All residues with a score > 0.5 were considered to significantly contribute to the binding free energy of the interaction and therefore assigned as a hotspot residue.

### Protein–RNA Hotspot Residues

Of the residues related to neurocognitive outcomes, only R57 was considered a hotspot residue in the interaction of Tat subtype B and TAR (Table 4). In Tat subtype C, R57 is mutated to S57. From the total interacting residues in the Tat subtype B-TAR (Table 2), Met1, Glu2, Asp5, Lys19, Cys22, Lys50, Ser46, Tyr47, Lys50, Lys51, Arg53, Gln54, Arg55, Arg56, and Arg57 were considered hotspot residues. This highlights the importance of the Arginine-rich region in Tat-TAR interaction in subtype B. Using Tat Subtype B as the positive control, from the hotspot residues, it is relevant to note that subtype C differs at position 40 with a K40Y mutation and 57 with R57S mutation. In subtype C interaction, Y40 and S57 are also considered hotspot residues.

## Discussion

In the current study, molecular modeling assisted in generating reliable Tat subtype B and subtype C protein structures useful for molecular docking studies. Furthermore, the docked complexes were validated using molecular dynamic simulations to determine stability, flexibility and free energy of binding between the TAR element and Tat subtype B and Tat subtype C protein structures, respectively. The docking results of this study showed that Tat subtype B had a higher binding affinity and number of interactions with the TAR element. Furthermore, the MD results demonstrated that Tat subtype B had more flexible regions and that the TAR element was less stable bound to Tat subtype B having fewer average number of hydrogen bonds with the TAR element. This reduced average number of hydrogen bonds between Tat-B and TAR is expected as the TAR element is less stable within the Tat-B binding site. This flexibility could account for the stronger affinity of the TAR element for Tat Subtype B based on the docking score and binding free energy values. Furthermore, the binding free energy values showed that the TAR element had a stronger affinity for Tat subtype B compared to Tat subtype C based on higher van der Waals energy and electrostatic energy contributions. The stronger binding might account for higher transactivation capacity of Tat subtype B compared to Tat subtype C. Additionally, the key neuropathogenic Tat protein signatures in subtype B (C31, R57, and Q63), residue R57 may be considered a key residue in stronger Tat-TAR binding. This may help explain why we see a higher level of neuropathogenesis, clinical prevalence, and severity of HAND in participants with subtype B infection.

To date, findings remain mixed, and it remains largely unclear as to which Tat subtype may yield higher binding affinity to the TAR RNA element and subsequent transactivation. Previous studies reported that Tat subtype C has a higher affinity for the TAR RNA element in a Tat-TAR electrophoretic mobility shift assay with subsequent stronger transactivation potentials (Desfosses et al., 2005; de Arellano et al., 2010; Bachu et al., 2012). Another study reasoned that Tat subtype C has a more flexible structure and this allows for a better and more stable binding to TAR (Johri et al., 2015). The inverse has also been argued reporting that Tat subtype C may have a relatively higher-ordered structure and be less flexible than Tat subtype B (Siddappa et al., 2006) thereby providing greater transactivation capacity (Siddappa et al., 2006; Ruiz et al., 2019). A recent study by Ronsard et al. (2017), has reported a greater binding affinity of Tat subtype B to TAR due to a greater number of H-bonds compared to Tat subtype C (Ronsard et al., 2017). Our findings build on these previous computational findings, as in addition to the docking of Tat subtypes to TAR done by Ronsard et al. (2017), we performed additional analyses including MDS analysis and the identification of protein–RNA hotspot residues. Here, we report a higher binding affinity of Tat subtype B to TAR as reported by a higher docking score, greater number of interactions (H-bonds and salt bridges) and higher binding free energy as indicated by higher van der Waals energy and electrostatic energy. The higher binding affinity of Tat subtype B to TAR is most likely due to Tat subtype B having more flexible regions and higher TAR flexibility when bound to Tat subtype B compared to Tat subtype C. Therefore, our findings are in alignment with previous computational and molecular studies suggesting a higher binding affinity of Tat subtype B to TAR and is suggestive of higher levels of transactivation (Siddappa et al., 2006; Ruiz et al., 2019). However, biochemical assays and binding studies will need to be performed to confirm the findings of the current study.

Furthermore, there is no clear consensus as to which subtype-specific Tat protein signatures may account for differential TAR interaction and subsequently transcription efficiency. A limited number of studies have investigated the influence of subtype-specific Tat mutations on TAR binding and transcriptional efficiency (Kurosu et al., 2002; Edwards et al., 2005; Ronsard et al., 2017). The Arginine residues at positions 52, 53, 55, 56, and 57 all contributed to the rigid TAR-Tat complex formation (Edwards et al., 2005). These findings were supported by a structural computational study that reported on Tat subtype B specific Lysine residues 28, 29, 50, 51, and 71 and Arginine residues 49, 52, 53, 55, 57, 58 were important residues for hydrogen bond formation with the TAR element (Ronsard et al., 2017). A study by Kurosu et al. (2002) reported that a Q63E mutation present in Tat subtype C contributed to greater transcriptional activation in human CD4 T cells. In our study, we found that the Arginine-rich region is crucial for TAR interaction in Tat subtype B-TAR complexes and in addition to this none of the residues within the Arginine-rich region (residues 48–58) showed high flexibility values, suggesting the active site is stable and not undergoing large conformational changes. For both Tat variants, residues in positions 51–73 were predicted to be disordered, supporting the premise that the residues of a protein that are able to bind RNA are intrinsically disordered (Ottoz and Berchowitz, 2020). Further, these findings are aligned with previous studies which suggest that the HIV-1 Tat protein is largely intrinsically disordered (Shojania and O'Neil, 2010; Kunihara et al., 2019). In this study, the slightly higher disordered state of subtype C may explain the weaker interaction with TAR. This further highlights the relevance of the Arginine domain in Tat-TAR interactions. In the Tat subtype B-TAR complex, Met1, Glu2, Asp5, Lys19, Cys22, Ser46. Tyr47, Lys50, Lys51, Arg53, Gln54, Arg55, Arg56, and Arg57 contributed to the largest electrostatic energy. From these energy important residues, Tat subtype C had mutations at residue 40 (K40T) and 57 (R57S). Considering that the presence of Lys40 and Arg57 are considered important in Tat subtype B-TAR interactions, we speculate that the change in these residues in Tat subtype C may contribute to a lower level of interaction between Tat subtype C with TAR. In a recent study, the Thr40 (76%) and S57 (74%) Tat signatures were highly prevalent in subtype C infected participants (de Almeida et al., 2021), and may have implications in Tat-TAR binding and other neuropathophysiological effects. Interestingly, in subtype C-TAR interactions, residues at position 40 (Thr) and 57 (Ser) were also considered as hotspot residues in the Tat subtype C-TAR interaction, which may suggest that even though these mutations may contribute to reduced binding of the TAR element compared to Tat subtype B, suggesting that these residues are important for the functioning of the Tat-TAR interaction complex. Therefore, these residues may be important in the viral transcription of HIV-1 and subsequently the pathogenesis of HIV-1. These key interacting residues may be investigated as targets in the development of therapeutics which inhibit interaction of the Tat-TAR complex.

In a previous review done by our group (Williams et al., 2020), we have highlighted key Tat protein signatures responsible for differential neuropathophysiology between Tat subtype B and subtype C. These included the C31S, R57S, and Q63E in Tat subtype C. We therefore investigated structurally if these mutations could potentially affect Tat-TAR binding. Our findings indicated that a mutation at position 57 in subtype B, may influence TAR binding. The R57 signature in Tat subtype B is crucial for transactivation, and transactivation by Tat subtype B was significantly reduced by the R57S substitution which is present in Tat subtype C (Ruiz et al., 2019). This may also explain why lower levels of neuropathogenesis are seen in PLWH with subtype C when compared to subtype B (Ruiz et al., 2019). We recently observed that the R57S mutation in South Africa participants accounted for lower levels of certain immune markers including C-C motif chemokine ligand 2 and thymidine phosphorylase (Williams et al., 2022) and these markers are related to neurocognitive impairment in PLWH (Ancuta et al., 2008; Cohen et al., 2011; Williams et al., 2019). Our findings suggest that the R57S mutation found in Tat subtype C may influence lower TAR binding and this may be an initiating step to why we see less severe neuropathophysiological (e.g., dysregulated inflammation) features related to HIV-1C infection (Ruiz et al., 2019). Future molecular studies should investigate these signatures as potential diagnostic markers of neuropathology in PLWH.

## Conclusion

In this study, we successfully generated accurate 3D structures for Tat subtype B and C using homology-modeling methods and found that each protein structure successfully satisfied all quality checks. Molecular docking studies indicated that Tat subtype B had a higher docking score and a great number of interactions with TAR compared to Tat subtype C. This is in agreement with the binding free energy calculations performed with the trajectory generated molecular dynamic simulations. The possible improved binding of TAR to Tat subtype B could be due to the increased flexibility of both Tat subtype B protein residues and the increased dynamic movement of the TAR element. Apart from all the key neuropathogenic Tat protein signatures, R57S present in Tat subtype C may contribute to the lower level of TAR interaction, transactivation, and underlying neuropathophysiology when compared to Tat subtype B. Future experimental studies should include binding studies and transcriptional assays to validate the role of Tat protein signatures in the development of neuropathology in PLWH.

## Data Availability Statement

The original contributions presented in the study are included in the article/Supplementary Material; further inquiries can be directed to the corresponding author.

## Author Contributions

MW conceptualized and designed the study and performed all computational experiments. RC performed the molecular dynamics simulations and contributed intellectually to the general concept of the manuscript and the flow of content. MW and RC reviewed the manuscript and suggested and made relevant changes before submission. All authors contributed to the article and approved the submitted version.

## Funding

The authors would like to acknowledge all funding contributors. MW was funded by DSI-NRF Research Development Grants for new Generation of Academics Programme (nGAP) Scholars and South African Society for Biological Psychiatry. RC was funded by the next Generation of Academics Programme (nGAP), Department of Higher Education and Training (DHET), South Africa.

## Conflict of Interest

The authors declare that the research was conducted in the absence of any commercial or financial relationships that could be construed as a potential conflict of interest.

## Publisher’s Note

All claims expressed in this article are solely those of the authors and do not necessarily represent those of their affiliated organizations, or those of the publisher, the editors and the reviewers. Any product that may be evaluated in this article, or claim that may be made by its manufacturer, is not guaranteed or endorsed by the publisher.

## Footnotes

^1^ https://www.uniprot.org/uniprot/P05905

^2^ https://www.uniprot.org/uniprot/O12161

^3^ http://bioinf.cs.ucl.ac.uk/psipred

^4^ https://swissmodel.expasy.org

^5^ https://saves.mbi.ucla.edu/

^6^ https://prosa.services.came.sbg.ac.at

^7^ http://sysbio.rnet.missouri.edu/3Drefine/

^8^ https://plip-tool.biotec.tu-dresden.de/plip-web/plip/index

^9^ http://denglab.org/PrabHot/
