# Supplementary material for: Molecular Modeling of Subtype-Specific Tat Protein Signatures to Predict Tat-TAR Interactions That May Be Involved in HIV-Associated Neurocognitive Disorders
Source: Front Microbiol. 2022 Apr 7;13:866611. doi: 10.3389/fmicb.2022.866611 (PMC9021916; doi:10.3389/fmicb.2022.866611)
Supplement: Supplementary file 1 [file Table_1.DOCX]

Supplementary Material

# Supplementary Figures


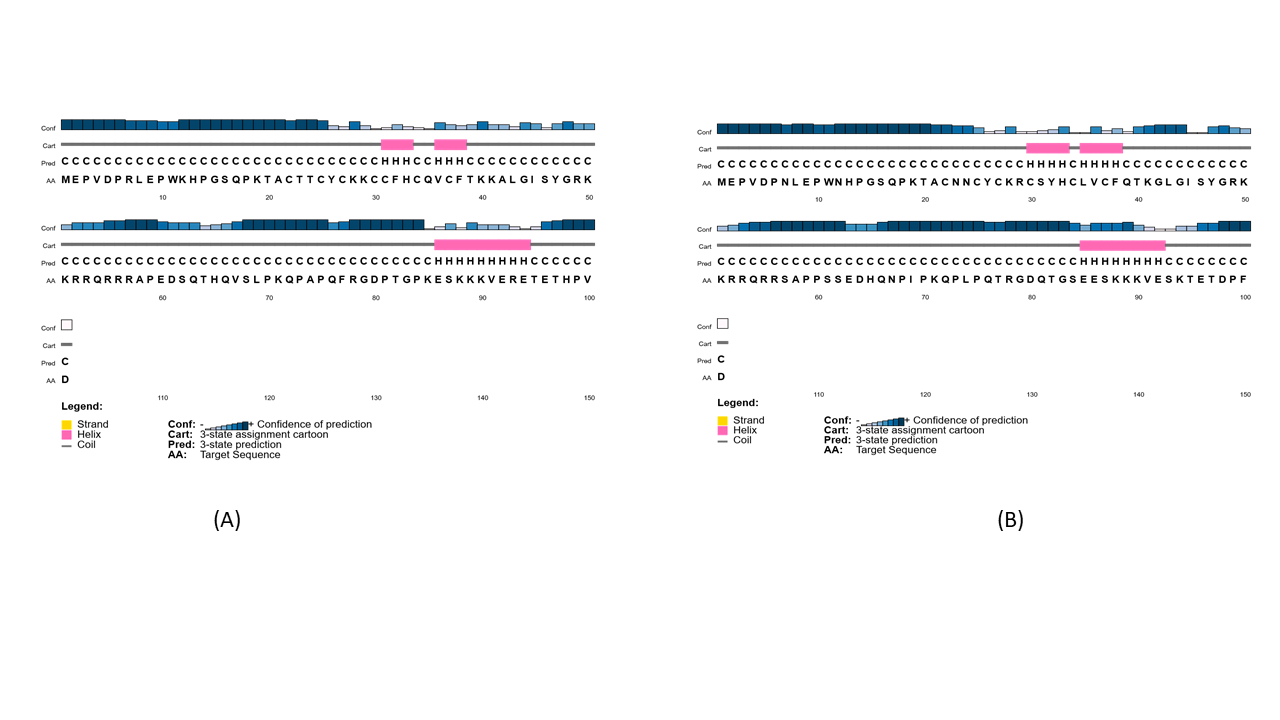


**Supplementary Figure 1 (A-B).** Secondary structure prediction of (A) Subtype Tat B and (B) Subtype Tat C from PSIPRED. Subtype B and C Tat proteins are predicted to contain 3 alpha helical structures respectively.


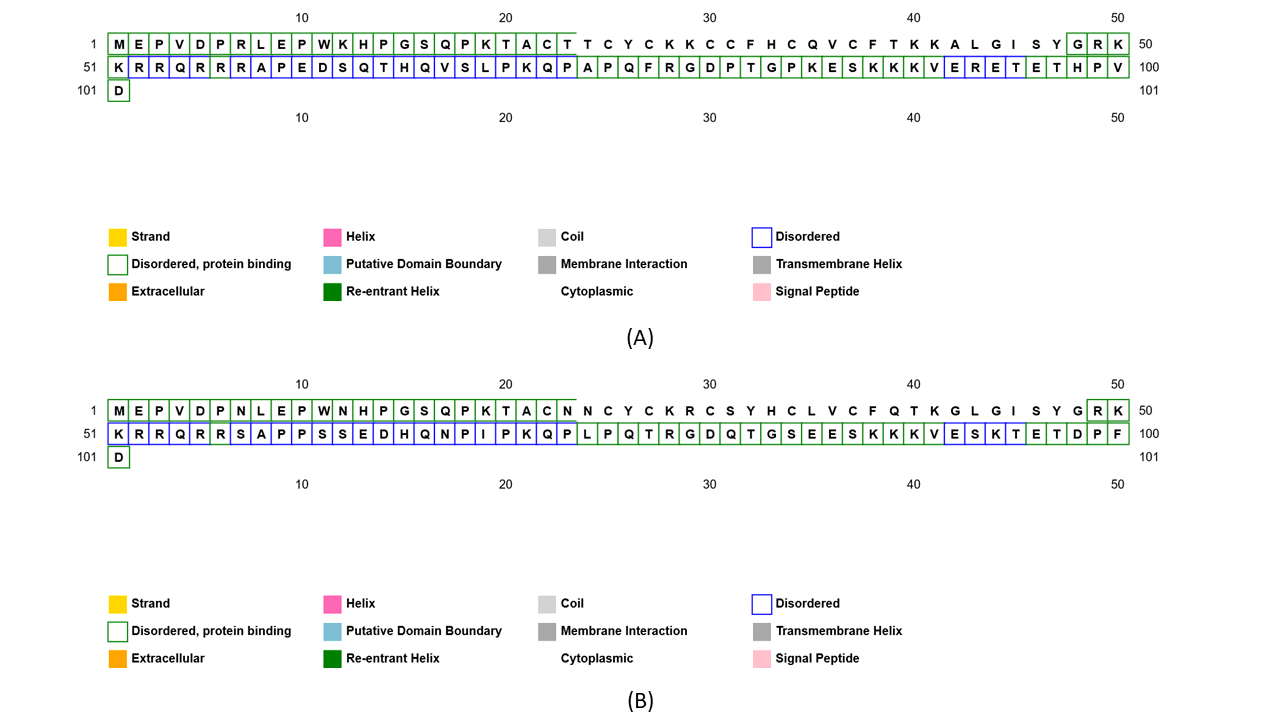


**Supplementary Figure 2 (A-B).** Prediction of disordered state of Tat variants (A) Subtype Tat B and (B) Subtype Tat C from DISOPRED3.


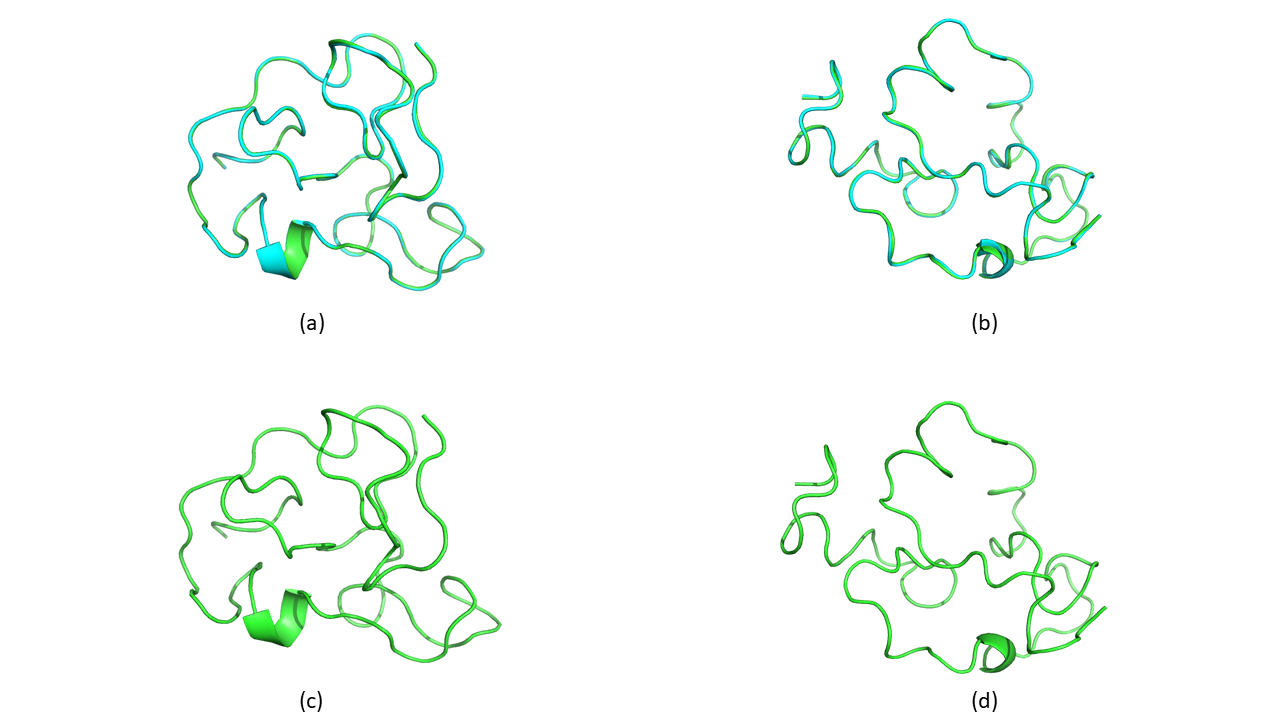


**Supplementary Figure 3 (A-B)** Superimposition of Tat subtype B (green) structure onto template 1jfw.1.A structure (cyan) (0.169Å) and (b) Superimposition of Tat subtype C (green) structure onto template 1tbc.1.A structure (cyan) (0.515Å).


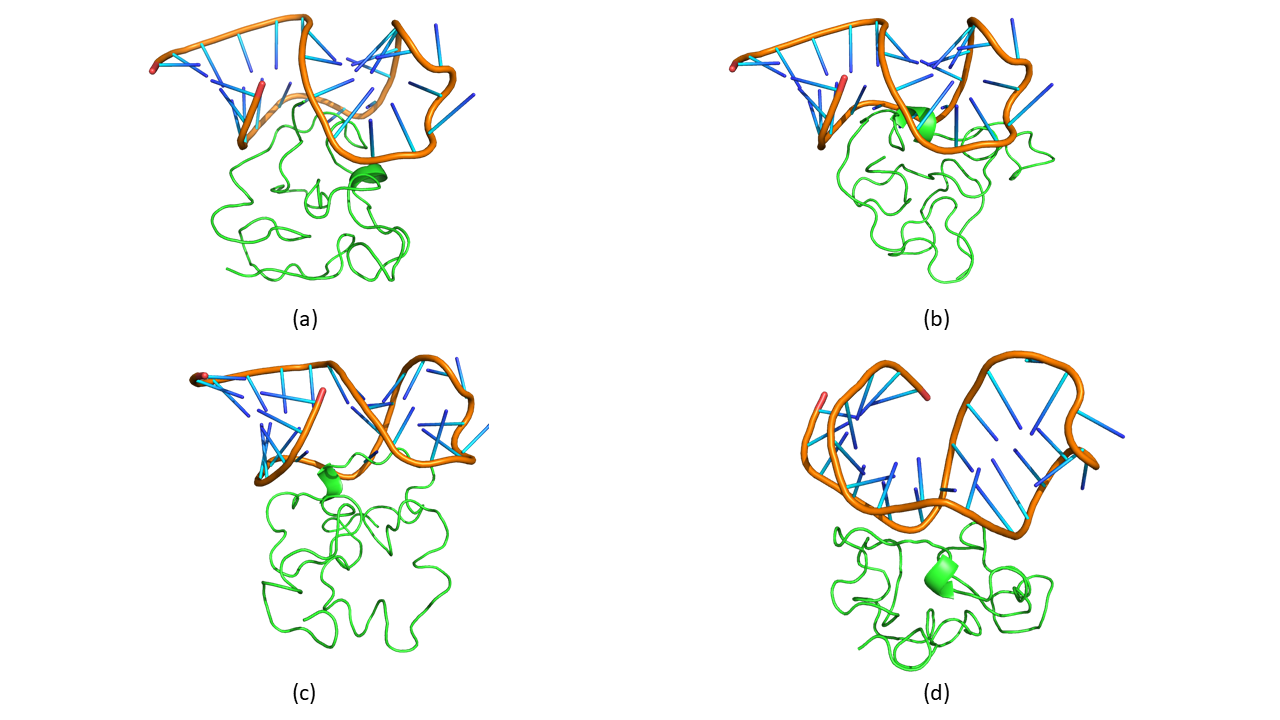


**Supplementary Figure 4 (A-D):** Selected active site vs blind docking. (A) Selected residues in active site of Tat subtype B docked against TAR and (B) blind docking of Tat Subtype B against TAR. (D) Selected residues in active site of Tat subtype C docked against TAR and (E) blind docking of Tat Subtype C against Tar.
